# Potential Utility of Oral Mucosal Capillaroscopy as an Indicator of Microvascular Damage in Behçet Disease: A Preliminary Study

**DOI:** 10.5826/dpc.1104a116

**Published:** 2021-10-01

**Authors:** Abdullah Demirbaş, Ömer Faruk Elmas, Gözde Ulutaş Demirbaş, Mustafa Atasoy, Ümit Türsen, Torello Lotti

**Affiliations:** 1 Department of Dermatology, Kütahya Health Sciences University; 2 Department of Dermatology, Kırıkkale University Kırıkkale, Turkey; 3 Department of Dermatology, Evliya Çelebi Training and Research Hospital, Kütahya, Turkey; 4 Health Science University, Kayseri City Hospital, Department of Dermatology, Kayseri, Turkey; 5 Mersin University, Department of Dermatology, Mersin, Turkey; 6 Department of Dermatology, Guglielmo Marconi University, Rome, Italy

**Keywords:** Behçet disease, capillary, handheld dermoscopy, mucoscopy, microvascular damage, oral labial mucosa

## Abstract

**Introduction:**

Behçet disease (BD) is an auto-inflammatory condition characterized by multisystemic vasculitis. Oral mucosal capillaroscopy is an easy-to-use, repeatable, non-invasive method for evaluating mucosal microvasculature, contributing to the differential diagnosis and prognosis of various acute and chronic inflammatory diseases.

**Objectives:**

This study aims to characterize and describe the oral labial mucosal capillary findings in patients with BD using handheld dermatoscopy and to investigate the relationship between the capillary findings and the severity of the disease.

**Methods:**

This cross-sectional study included patients with BD and healthy subjects. Capillaroscopic examination of the oral labial mucosa in each subject was performed by a handheld dermatoscope using polarized light. The clinical severity of BD was evaluated using Krause’s Clinical Severity Scoring for BD.

**Results:**

Sixty patients with BD and 60 healthy subjects were enrolled in the study. The frequencies of irregular capillaries, microhemorrhages, glomerular vessels, megacapillaries, and tortuous vessels were statistically significantly higher in the patient group when compared to the healthy individuals. In addition, a correlation was detected between the oral mucosal capillaroscopic findings and disease duration, severity, and vascular complications.

**Conclusions:**

Our study is the first to explore the potential role of oral mucosal capillaroscopic examination in patients with BD. Data obtained from this study indicated that oral mucosal capillaroscopy may be a useful tool to demonstrate microvascular damage in patients with BD.

## Introduction

Capillaroscopy is a non-invasive diagnostic technique used to image the peripheral circulation in autoimmune diseases and acute and chronic inflammatory diseases. Capillaroscopic examinations aim to investigate microvascular damage, which is directly related to many clinical findings both during diagnosis and follow-up [1–3]. Oral mucosa capillaroscopy is an easyto- use, repeatable, non-invasive, and well-tolerated method that evaluates the mucosal microvasculature and contributes to the differential diagnosis and prognosis of various autoimmune disorders. The visualization and evaluation of the microvascular bed are simpler to perform at the level of the oral mucosa than on cutaneous surfaces, because of the absence of stratum corneum in some parts of the oral mucosa [4–8].

Behçet disease (BD) is a systemic auto-inflammatory disease characterized by 4 main features; recurrent oral aphthous ulcers, genital ulcers, ocular involvement, and skin lesions. The underlying etiopathogenesis of the disease has not been fully elucidated yet. It is believed that the disease may be triggered by some external factors in individuals with a genetic predisposition and thus, it may cause an autoimmune response by activating the immune system. This results in multisystemic vasculitis, which is considered the key pathogenetic factor in BD. BD may involve any kind and size of arterial and venous vessels, therefore it may present with various clinical pictures. For instance, larger vascular involvements are considered as a a poor prognostic indicator. Therefore, early diagnosis of microvascular damage is important to prevent these silent and potentially fatal vascular complications and to prepare an appropriate treatment plan [9–16].

This study aims to describe the oral labial mucosal capillary findings in patients with BD, using dermoscopy, and to investigate the relationship of capillary findings with the duration and severity of the disease.

## Materials and Methods

### Subjects

This research was designed as a single center, cross-sectional study. The study included patients with Behcet Disease and age-gender matched healthy volunteers. Sixty BD patients and 60 control patients admitted to our outpatient clinic between June 1, 2020, and September 1, 2020, were included in the study (all subjects were Caucasian).

The parameters considered for each patient included disease duration (years), history of medication, oral ulcers, genital ulcers, erythema nodosum, superficial thrombophlebitis, acneiform eruption, arthralgia, arthritis, deep vein thrombosis, arterial involvement, ocular involvement, neurological involvement, gastrointestinal involvement, genitourinary involvement, large vessel involvement, pathergy positivity, clinical severity score, and HLA-B51 status.

### Disease Severity Assessment

The severity of the disease was evaluated using Krause’s Clinical Severity Scoring for BD (The total score is obtained by giving one point for each mild symptoms such as oral aphthae, genital ulcer, arthralgia, erythema nodosum, papulopustular lesions, folliculitis; two points for the moderate symptoms such as arthritis, deep-vein thrombosis, anterior uveitis; and 3 points for each severe symptom such as posterior uveitis/panuveitis, retinal vasculitis, arterial thrombosis, neuro-Behcet, and bowel perforation). Based on the total score, the patients were sub-classified into 3 groups as mild (<4), moderate (4–6), and severe (≥7) [17].

### Inclusion and Exclusion Criteria

All patients with BD met the criteria of the International Behçet Disease Study Group [18]. During the examination process, all subjects had intact oral mucosa.

Exclusion criteria were as follows; oral mucosal pathologies (candidiasis, lichen planus, glossitis, periodontitis, etc.), patients under 18 years of age, smoking, the use of medications that may disrupt the microvascular structure (antidiabetics, antihypertensives, antilipidemics and thyroid medicines), pregnancy, breastfeeding, and systemic disorders such as systemic vasculitis, scleroderma, SLE, dermatomyositis, Sjögren syndrome, antiphospholipid syndrome, mixed connective tissue disease, diabetes mellitus, hyperthyroidism, hypothyroidism, hypertension, and cardiovascular diseases.

### Oral Mucosa Capillaroscopy

Labial mucosal capillaroscopic examination was performed by 10-times hand-held dermatoscope using polarized light (DermLite DL4, 3gen Inc, CA, USA). Capillaroscopic images were acquired using a high-resolution cell phone attached with a 2 × optical zoom (iPhone 11, Apple Inc, CA, USA). A total of 20 times magnification was achieved. Contact dermoscopic examination without fluid was performed with the same light source, at the same room temperature (24 ° C), by the same operator in the morning. To standardize measurements and avoid artifacts, each subject was seated while examination was ongoing and each area was examined twice.

The examined area was the lower lip mucosa for each patient. The capillary morphology was defined using internationally accepted dermoscopic terminology [19]. The images obtained were evaluated by a capillaroscopic examination specialist.

### Statistical Analysis

Descriptive statistics of the obtained data were calculated as mean ± standard deviation (SD), quartiles (25th, median, and 75th), number and percentage frequencies depending on the type of features. Compliance of numerical properties to normal distribution was examined by the Kolmogorov-Smirnov test. Independent samples t test was used to compare the patient and control groups in terms of age, and the Pearson chi-square test was used in terms of oral capillaroscopic findings. Also, the relationships between patient characteristics and oral capillaroscopic findings were evaluated using Pearson’s chi-square test. Statistical significance level was accepted as p <0.05 and SPSS (ver. 23, SPSS Inc, Chicago, IL, USA) program was used in calculations.

### Ethics Approval

All the procedures followed the Helsinki declaration, and the study was approved by an Institutional Review Board (Decision date and number:2020/025). Written informed consent was obtained from all participants.

## Results

### Demographic and Clinical Features

A total of 60 patients with BD (27 females, 33 males) and 60 (28 females, 32 males) age and gender-matched healthy controls were included in the study. The mean age of patients and controls was 34.97±9.02 and 34.75±6.41, respectively (p = 0.880). The mean disease duration was 8.608 ± 7.5504 years. The mean clinical severity score was 5.73 ± 2.577. According to the clinical scores, 9 (15%) patients were considered to have mild disease, 33 (55%) moderate disease, and 18 (30%) severe disease. All patients were on at least 1 systemic treatment for BD. The most common treatment used was colchicine monotherapy (n=37,61.7%) followed by azathioprine monotherapy (n=9, 15%). The most common combined regimen was composed of colchicine and azathioprine (n=7, 11.67%). Combinations of colchicine and corticosteroid (n=5, 8.3%), and azathioprine and corticosteroid (n=2, 3.33%) were the other regimens used (Table 1).

The most common clinical manifestation of BD was recurrent oral aphthae (n=60,100%), followed by recurrent genital ulcer in (n=48, 80%), erythema nodosum (n=23, 8.3%), superficial thrombophlebitis (n=5, 8.3%), acneiform eruption (n=48, 80%), ocular involvement (n=22, 36.7%), arthralgia (n=46, 76.7%), arthritis (n=16, 26.7%), pathergy positivity (n=21, 35%), deep vein thrombosis (n=6, 10%), large vessel involvement (n=5, 8.3%), genitourinary involvement (n=3, 5%), arterial involvement (n=2, 3.3%), neurological involvement (n=1, 1.7%), and gastrointestinal involvement (n=1, 1.7%). Eleven patients (18.3%) showed HLA B51 positivity (Table 2).

### Capillaroscopic Features

**Figure 1:**
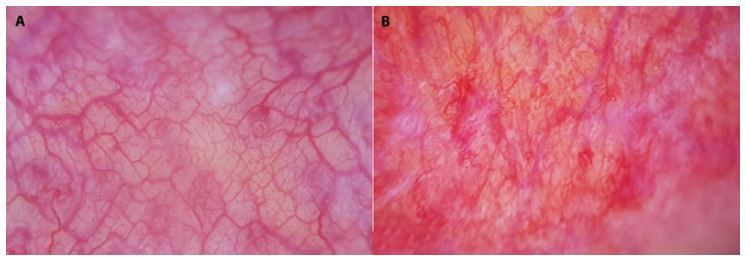
(A) Regular capillaries. (B) Irregular capillaries.

**Figure 2:**
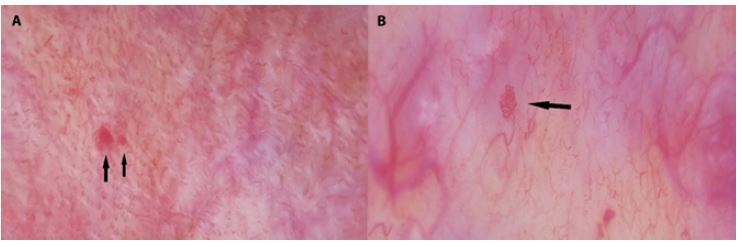
(A) Microhemorrhage (black arrows). (B) Glomerular vessel (black arrow).

**Figure 3:**
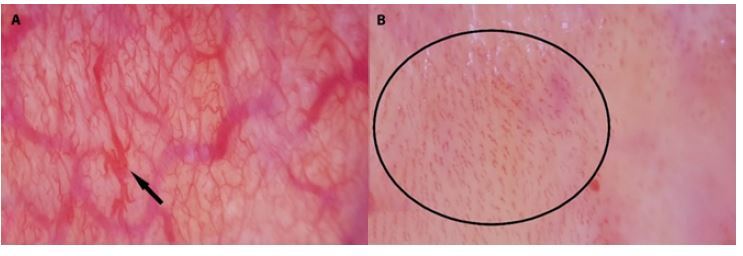
(A) Megacapillar (black arrow). (B) Tortuous vessels (circled area).

**Figure 4:**
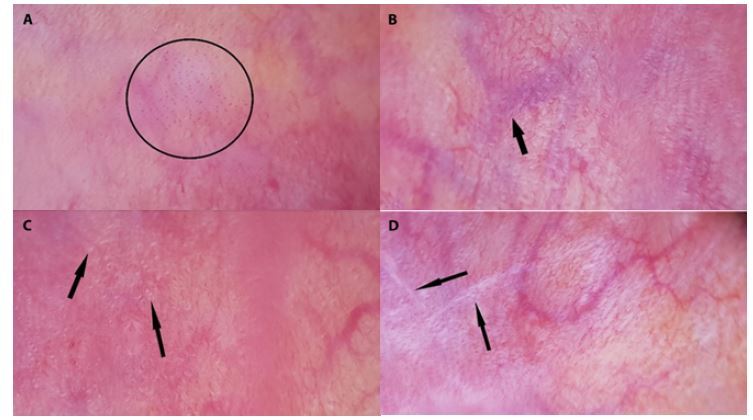
(A) Dotted vessels (circled area). (B) Purple areas (black arrow). (C) White dots (black arrows). (D) Hyperkeratosis (black arrows).

The frequency of irregular capillaries (Figure 1, A and B), microhemorrhage (Figure 2A), glomerular vessels (Figure 2B), megacapillaries (Figure 3A), and tortuous vessels (Figure 3B) were statistically significantly higher in patients with BD when compared to the healthy subjects (P < 0.001, P = 0.049, P < 0.001, P <0.001, P< 0.001 respectively). There was no statistically significant difference between the two groups in terms of dotted vessels (Figure 4A), purple area (Figure 4B), white dots (Figure 4C) and hyperkeratosis (Figure 4D) (P = 0.584, P= 0.465, P = 0.609, P = 0.591, respectively) (Table 3).

Statistically significant correlations were found between erythema nodosum, superficial thrombophlebitis, HLAB51 and microhemorrhage, glomerular vessels, and megacapillaries (P < 0.05). There were also statistically significant correlations between irregular capillaries and erythema nodosum and acneiform rash (P < 0.05). The presence of irregular capillaries, microhemorrhages, glomerular vessels, megacapillaries, deep vein thrombosis and major vascular involvement showed statistically significant correlations (P < 0.05). No statistically significant correlation was detected between dotted vessels, purple area, white dots, hyperkeratosis, and mucocutaneous, systemic and vascular manifestations of BD (P < 0.05) (Tables 4 and 5).

The clinical severity score and disease duration were found to be significantly higher in patients with irregular capillaries, microhemorrhage, glomerular vessels, megacapillaries, and tortuous vessels (P < 0.05) (Table 6).

## Discussion and Conclusions

This preliminary study highlights the potential utility of oral capillaroscopy in detecting microvascular findings in patients with BD.

BD is a chronic, systemic vasculitis that may involve any kind and size of vessels, and is characterized by recurrent oral aphthous ulcers, genital ulcers, skin lesions, and ocular inflammation. Although the underlying etiopathogenetic mechanisms of the disease have not been fully elucidated, it is suggested that an abnormal immune response occurs in individuals with genetic predisposition due to the antigenic effect of environmental factors, particularly infectious agents. This abnormal immune response causes endothelial dysfunction and vascular damage, which is thought to be associated with potentially life-threatening complications for BD. In this context, early diagnosis of microangiopathic damage becomes important to prevent such vascular complications and to set an appropriate treatment plan [9–16].

Capillaroscopy is a non-invasive diagnostic technique that allows the imaging of the peripheral vascular structures and is used to determine microangiopathic damage at the early stages. Devices such as video-capillaroscopy and dermoscopy are used for capillaroscopic examination. The video-capillaroscope is the most sophisticated device and provides a detailed examination. However, since it is an expensive device, it is not available in many centers. Today, there are studies conducted using hand-held dermatoscopes or computed dermatoscopes. These studies reported positive results, further supporting the importance of capillaroscopy examination via dermatoscopy. Studies comparing video-capillaroscope and dermoscopy, show that dermoscopy is as effective as video-capillaroscope [20–23].

For a long time, proximal nail fold was the preferred location for capillaroscopic examination [1,2,24,25]. However, recent studies demonstrate that capillaroscopy of oral microcirculation is well-tolerated, easy, fast, and provides excellent visibility of microcirculation. The absence of stratum corneum in some parts of oral mucosa allows more clear visualization of the microvascular bed. Therefore, oral mucosa capillaroscopy has been the subject of many studies investigating acute and chronic systemic inflammatory diseases. In these studies, vascular visibility in the labial mucosa was suggested to be the best location of the oral region [4–8].

Examination of the microvasculature with proximal nailfold capillaroscopy has been shown to be effective in determining early morphological markers of microvascular damage that are useful in the early diagnosis of systemic rheumatic diseases [26,27]. The formation mechanism of capillary morphological changes, which are common in connective tissue diseases, has been defined in conducted studies. Megacapillaries develop as an abnormal angiogenic response to peripheral ischemia and are accepted as the first sign of microvascular damage. Besides, capillary microhemorrhage indicating ischemia-reperfusion injury has been described. Tortuous vessels have been reported to occur secondary to hypoxic status in patients with advanced vascular damage [26–28]. Some studies suggested that proximal nailfold capillaroscopy is also effective in determining the degree of vascular damage in BD [29–31]. In BD, the most important factor causing changes in capillary morphology is shown to be endothelial dysfunction. It was stated that patients with longer disease duration and higher severity should have more severe damage to the capillaries. However, in the proximal nailfold capillaroscopy study, correlations between disease duration and microhemorrhage and megacapillary features were found to be weak. Besides, no significant relationship between capillary morphological changes and disease activity and severity was shown [32].

In our study, we detected significant relationships between disease duration, severity, and oral mucosa capillaroscopic findings such as irregular capillaries, microhemorrhage, glomerular vessels, megacapillary, and tortuous vessels.

In 2 different oral mucosa capillaroscopy studies conducted by Scardina et al, they found that patients with rheumatoid arthritis had less calibered and elongated vessels compared to the healthy controls and that microvascular changes were correlated with disease activity [33,34]. Another study showed that patients with systemic sclerosis had fewer, large-diameter, and curled capillaries compared to controls and that microvascular changes were correlated with disease activity. Based on these findings, it has been suggested that capillary changes due to systemic sclerosis are not only limited to the nailfold but also occur in the oral mucosal microcirculation [35]. In a study conducted on patients with diabetic foot, a reduction in capillary density and length; and an enhancement in capillary curling were observed. The authors emphasized that oral mucosa capillaroscopy has an important diagnostic role in the early detection of microangiopathic damage [36].

Scardina et al, showed that patients with Burning mouth syndrome had increased capillary diameter compared to the healthy controls. The etiopathogenesis of Burning mouth syndrome is not clear but vascular involvement is thought to play a role [37]. In a study conducted on 25 patients with head and neck tumors to evaluate the effect of cytotoxic chemotherapy on oral mucosal microvascularity; a significant change in the labial capillary vessels was reported [38]. In a study investigating the long-term effects of smoking on oral mucosa capillaries; capillary changes have been shown to persist for a long time even if the individual quits smoking [39]. Another study showed that microvascular disorders including hemorrhagic dots, matchstick hairpin vessels, and microaneurysm are more frequent in smokers compared to non-smokers [40].

Our study is not devoid of limitations. The small sample size of patients and controls at a single clinic may not reflect the general population. The cross-sectional design of the study does not allow to know whether oral mucosa capillary findings are associated with the long-term complications. Therefore, long-term prospective studies with larger sample size are needed. In addition, it is hard to be sure that the findings we observed are not directly associated with the drugs used by the examined patients, as all enrolled patients were undergoing at least 1 systemic medication. Another limitation of our study is the lack of a group of patients with the conditions to cause capillary morphological findings, such as diabetes, hypertension, and smoking habits. Hence, it is not possible to determine how specific our findings were for BD in real terms. Finally, morphometric measurements of the detected capillary structures could not be made due to the use of handheld dermoscopy.

To sum up, our study is the first to explore the potential role of oral mucosal capillaroscopic examination in patients with BD. We found a statistically significant difference in the presence of irregular capillaries, microhemorrhages, glomerular vessels, megacapillaries, and tortuous vessels in patients with BD compared to healthy subjects. We also observed a significant relationship between these capillaroscopic findings and vascular complications, disease duration, and clinical severity score. Larger and prospective studies are needed to determine whether oral mucosal microvascular changes can be used as an early predictor of serious BD complications.

## Figures and Tables

**Table 1:** Demographic, Clinical, and Treatment Characteristics of Behçet Disease Group and Control Group

	Behçet Patients (N=60)	Controls (N=60)	P-value

**Gender, N (%)**			0.855
**Male**	33 (%55)	32 (%53.3)	
**Female**	27 (%45)	28 (%46.7)	

**Age, Mean±Sd**	34.97 ± 9.02	34.75 ±6.41	0.880

**Disease duration (years), Mean±Sd**	8.608 ± 7.5504	-	-

**Clinical Severity Score, Mean±Sd**	5.73 ± 2.577	-	-

**Disease Activity, N (%)**			
**Mild**	9 (%15)	-	-
**Moderate**	33 (%55)		
**Severe**	18 (%30)		

**Medications, N (%)**			
**Azathioprine**	9 (%15)	-	-
**Azathioprine + Corticosteroid**	2 (%3.33)		
**Colchicine**	37 (%61.7)		
**Colchicine + Azathioprine**	7 (%11.67)		
**Colchicine + Corticosteroid**	5 (%8.33)		

Sd= Standard deviation

**Table 2:** Clinical Symptoms of Behçet Disease Group

Symptoms	N (%)

Mouth Ulceration	60 (%100)
Genital Ulceration	48 (%80)
Erythema Nodosum	23 (%38.3)
Acneiform Rash	48 (%80)
Superficial Thrombophlebitis	5 (%8.3)
Deep-vein Thrombosis	6 (%10)
Arthralgia	46 (%76.7)
Arthritis	16 (%26.7)
Eye Involvement	22 (%36.7)
Neurological Involvement	1 (%1.7)
Gastrointestinal Involvement	1 (%1.7)
Genitourinary System	3 (%5)
Major vessel Involvement	5(%8.3)
Arterial Involvement	2 (%3.3)
Positive Pathergy Test	21 (%35)
Positivity of HLA B51	11 (%18.3)

**Table 3:** Distribution of the Oral Mucosa Capillaroscopy Findings of Behçet Disease Group and Control Group

Capillaroscopic findings	Behçet patients (n=60) N (%)	Control group (n=60) N (%)	P-value

**Capillary regularity**			
**Regular**	36 (%60)	60 (%100)	<**0.001**
**Irregular**	24 (%40)	0 (%0)	

**Dot vessels**	32 (%53.3)	29 (%48.3)	0.584

**Microhemorrhage**	14 (%23.3)	6 (%10)	**0.049**

**Glomerular vessels**	22 (%36.7)	0 (%0)	<**0.001**

**Megacapillary**	23 (% 38.3)	4 (%6.7)	<**0.001**

**Tortuous vessels**	54 (%90)	4 (%6.7)	<**0.001**

**Purple areas**	28 (%46.7)	32 (%53.3)	0.465

**White dots**	10 (%16.7)	8 (%13.3)	0.609

**Hyperkeratosis**	7 (%11.7)	9 (%15)	0.591

P < 0.05 is defined statistically significant and shown in bold.

**Table 4:** Comparison of the Relationship Between Mucocutaneous Symptoms and Oral Mucosa Capillaroscopic Findings in Behçet Patients

Symptoms	Capillary regularity		Microhemorrhage		Glomerular vessels		Megacapillary		Tortuous vessels	

	Irregular capillary (N=24)	Regular capillary (N=36)	+ (N=14)	− (N=46)	+ (N=22)	− (N=38)	+ (N=23)	− (N=37)	+ (N=54)	− (N=6)

**Genital Ulceration +**	20 28		12 36		19 29		19 29		44 4	
**−**	4 8		2 10		3 9		4 8		10 2	
**p**	(P=0.598)		(P=0.542)		(P=0.348)		(P=0.690)		(P=0.389)	

**Erythema Nodosum** +	16 7		10 13		13 10		16 7		22 1	
**−**	8 29		4 33		9 28		7 30		32 5	
**p**	**(P< 0.001)**		**(P=0.004)**		**(P=0.012)**		**(P=0.001)**		(P=0.250)	

**Acneiform Rash +**	23 25		13 35		21 27		22 26		44 4	
**−**	1 11		1 11		1 11		1 11		10 2	
**p**	**(P=0.012)**		(P=0.170)		**(P=0.023)**		**(P=0.017)**		(P=0.389)	

**Superficial +**	3 2		3 2		4 1		4 1		5 0	
**Thrombophlebitis −**	21 34		11 44		18 37		19 36		49 6	
**P**	(P=0.340)		**(P=0.043)**		**(P=0.036)**		**(P=0.045)**		(P=0.436)	

**Pathergy Test +**	11 10		7 14		9 12		10 11		21 0	
**−**	13 26		7 32		13 26		13 26		33 6	
**P**	(P=0.151)		(P=0.179)		(P=0.465)		(P=0.278)		**(P=0.050)**	

**HLA B51 +**	7 4		5 6		6 5		7 4		10 1	
**−**	17 32		9 40		16 33		16 33		44 5	
**P**	(P=0.077)		**(P=0.050)**		(P=0.173)		**(P=0.050)**		(P=0.911)	

+ = Present, − = Absent

P < 0.05 is defined statistically signifcant and shown in bold.

**Table 5:** Comparison of the Relationship Between Systemic and Vascular Clinical Findings and Oral Mucosa Capillaroscopic Findings in Behçet Patients

Symptoms	Capillary regularity		Microhemorrhage		Glomerular vessels		Megacapillary		Tortuous vessels	

	Irregular capillary (N=24)	Regular capillary (N=36)	+ (N=14)	− (N=46)	+ (N=22)	− (N=38)	+ (N=23)	− (N=37)	+ (N=54)	− (N=6)

**Deep−vein +**	6 0		5 1		6 0		6 0		6 0	
**Thrombosis −**	18 36		9 45		16 38		17 37		48 6	
**P**	**(P=0.002)**		**(P=0.001)**		**(P=0.001)**		**(P=0.001)**		(P=0.389)	

**Arthralgia +**	22 24		12 34		19 27		21 25		44 2	
**−**	2 12		2 12		3 11		2 12		10 4	
**P**	**(P=0.025)**		(P=0.361)		(P=0.177)		**(P=0.035)**		**(P=0.008)**	

**Arthritis +**	9 7		7 9		9 7		10 6		16 0	
**−**	15 29		7 37		13 31		13 31		38 6	
**P**	(P=0.121)		**(P=0.024)**		**(P=0.050)**		**(P=0.020)**		(P=0.119)	

**Eye Involvement +**	10 12		8 14		12 10		10 12		22 0	
**−**	14 24		6 32		10 28		13 25		32 6	
**P**	(P=0.512)		(P=0.069)		**(P=0.029)**		(P=0.388)		**(P=0.049)**	

**Neurological +**	1 0		1 0		1 0		1 0		1 0	
**Involvement −**	23 36		13 46		21 38		22 37		53 6	
**P**	(P=0.217)		(P=0.068)		(P=0.185)		(P=0.201)		(P=0.737)	

**Gastrointestinal +**	1 0		1 0		1 0		1 0		1 0	
**Involvement −**	23 36		13 46		21 38		22 37		53 6	
**P**	(P=0.217)		(P=0.578)		(P=0.185)		(P=0.201)		(P=0.737)	

**Genitourinary +**	2 1		3 0		3 0		3 0		3 0	
**System −**	22 35		11 46		19 38		20 37		51 6	
**P**	(P=0.333)		**(P=0.001)**		**(P=0.020)**		**(P=0.024)**		(P=0.554)	

**Major vessel +**	5 0		4 1		5 1		5 1		5 0	
**Involvement −**	19 36		10 45		17 37		18 36		49 6	
**P**	**(P=0.004)**		**(P=0.002)**		**(P=0.002)**		**(P=0.003)**		(P=0.436)	

**Arterial +**	2 0		2 0		2 0		2 0		2 0	
**Involvement −**	22 36		12 46		20 38		21 37		52 6	
**P**	(P=0.078)		**(P=0.009)**		**(P=0.050)**		(P=0.068)		(P=0.632)	

+ = Present, − = Absent

P < 0.05 is defined statistically signifcant and shown in bold, Standard deviation

**Table 6:** Comparison of the Relationship Between Disease Duration and Clinical Severity Score and Oral Mucosa Capillaroscopic Findings in Patients With Behçet Disease

Capillaroscopic Findings (N)	Disease Duration Mean±Sd	Clinical Severity Score Mean±Sd

**Capillary regularity Regular (36)**	7.4 ± 6.7	5±1
**Irregular (24)**	10.4 ± 8.5	8 ± 3
**P**	(P=0.104)	**(P=0.001)**

**Dot vessels + (32)**	10.1 ± 8.5	7± 3
**− (28)**	6.9 ± 6	5±1
**P**	(P=0.174)	**(P=0.005)**

**Microhemorrhage + (14)**	14.4 ± 9.1	9±3
**− (46)**	6.9 ± 6.1	5± 1
**P**	**(P=0.001)**	**(P=0.001)**

**Glomerular vessels + (22)**	12.6 ± 8.2	8± 3
**− (38)**	6.3 ± 6.1	4±1
**P**	**(P=0.001)**	**(P=0.001)**

**Megacapillary + (23)**	11.7 ± 8.5	8±3
**− (37)**	6.7 ± 6.3	4± 1
**P**	**(P=0.007)**	**(P=0.001)**

**Tortuous vessels + (54)**	9.1 ± 7.7	6± 3
**− (6)**	4 ± 4	3±0
**P**	**(P=0.048)**	**(P=0.001)**

**Purple areas + (28)**	8.3 ± 7.6	6±2
**− (32)**	8.9 ± 7.6	5±3
**P**	(P=0.651)	**(P=0.003)**

**White dots + (10)**	8.6 ± 7.6	6 ± 3
**− (50)**	8.6 ± 7.6	6± 2
**P**	(P=0.874)	(P=0.521)

**Hyperkeratosis + (7)**	9.1 ± 8.6	6± 4
**− (53)**	8.5 ± 7.5	6 ± 2
**P**	(P=0.822)	(P=0.752)

+=Present, − = Absent, P < 0.05 is defined statistically signifcant and shown in bold.

Sd= Standard deviation.
